# Sun-Baths

**Published:** 1870-07

**Authors:** 


### SUN-BATHS.

Ever take one? They are deliciously invigorating. Every family that is able to have a water bathing establishment in their dwelling, should provide a sun-bath also. All that is necessary is to have a small room next the roof of your house, with a sky light the size of the ceiling and one end of the room. Have this in a locality that will give you the sun for the greatest part of the day. In this room the patient can sit or lie down for fifteen or twenty minutes, exposing the bare skin to the full sunlight.

Sun-baths cost nothing, but are decidedly the most refreshing and life-giving baths one can indulge in, whether he be sick or well. Why, every housekeeper can tell you of the necessity for exposing woolen fabrics to the sun after they have been piled away in the closets for a season! We all know of the injury our clothes suffer from dampness, but never once think that an occasional exposure of our bodies to the sunlight is equally necessary to our health.

It is a great misfortune that these sun-baths cost nothing, for people are for ever deluded with the idea that those things only can be good or useful which cost money; they will cheerfully pay for Turkish or Russian baths, when they can secure far more beneficial ones for nothing, or for the trouble only of removing their raiment and exposing their bodies to the sun-light. How fortunate for God's creatures, both rich and poor, that the three things most necessary to good health,—sun-light, fresh air and pure water, are entirely *free* to all! You can have these blessed luxuries in abundance, without money and without price if you only will. If you would enjoy good health, then see to it that you are supplied with pure air to breathe, night and day; that you bathe for an hour or so every day, in the sun-light; and that you quench your thirst with no other fluid than water.

Let any invalid who reads this, and who has been housed for some time, take an occasional walk in the sun, even if it should only extend as far as the piazza, and let him observe the effect upon him—he will find it one of the most healthful baths he has ever taken.

Sleeping rooms should always be selected in such parts of the house as have the most benefit from the rays of the sun; the bed and bed-clothes should be thoroughly aired and kept in the sun as long as possible, every day. Many of the sleeping rooms in our Hotels are so situated as never to feel the influence of the sun's rays, and those who occupy such rooms for any length of time, will certainly suffer in health and strength.

The Italians have a proverb which says, "Where the sun does not enter, the Doctor must;" and with them the first point to be considered in the selection of a house is: What is its exposure to the sun? and they are always careful to locate their sleeping rooms on the side of the house most exposed to the rays of the sun.

Again, too many houses in our cities and villages are completely buried from the sun under the thick foliage of our shade trees. Elegant establishments these houses, whose occupants can command every luxury within the reach of wealth; saloons into which rank, beauty and fashion are welcomed, but from which the sunlight of Heaven is totally excluded by the shade trees.

At the risk of being denounced as an iconoclast, we would apply the axe to the root of at least two-thirds of the shade trees which surround the dwellings and line the streets of our city.

Let us have more sun-light and better health.

### OLD FOLKS.

A Rhode Island paper states that there is now living in the town of Smithfield, in that State, a man and his wife, Jonathan and Saloma Buxton, he being one hundred and two years old, and his wife one hundred and one, both enjoying perfect health, and able to attend to the duties and management of quite a large farm. They have now living nine children, the eldest seventy-six years of age, and all enjoying a remarkably youthful appearance. The old gentleman and his wife have enjoyed an unclouded matrimonial life of seventy-eight years, he never having had to call the services of a physician since his remembrance. His father, being one of the early emigrants from England, named the town Smithfield, and the street Buxton Street after the place he left in England.—*Med. Record.*

